# GSTA1 deficiency drives neuroendocrine differentiation via TNFRSF13B/c-FOS/CHGA axis in prostate cancer

**DOI:** 10.7150/ijbs.120497

**Published:** 2026-01-14

**Authors:** Jiajun Qian, Yang Luo, Yao Fu, Wenli Diao, Qiubo Ding, Wei Chen, Xuefeng Qiu, Hongqian Guo

**Affiliations:** 1 Department of Urology, Affiliated Drum Tower Hospital, Medical School of Nanjing University, Nanjing 210008, China.; 2 Institute of Urology, Nanjing University, Nanjing 210008, China.; 3 Department of Pathology, Affiliated Drum Tower Hospital, Medical School of Nanjing University, Nanjing 210008, China.

**Keywords:** prostate cancer, neuroendocrine differentiation, glutathione S-transferase alpha 1, androgen deprivation therapy

## Abstract

**Rationale:** Androgen deprivation therapy (ADT) is the cornerstone of prostate cancer (PCa) treatment. Prolonged ADT inevitably increases the risk of neuroendocrine differentiation, which leads to the development of hormone-refractory subtypes. In this study, we explored the molecular mechanisms underlying the neuroendocrine differentiation of PCa cells under ADT.

**Methods:** We performed digital spatial profiling (DSP) sequencing using tissue microarrays from five patients with PCa who underwent neoadjuvant therapy before radical prostatectomy at the Nanjing Drum Tower Hospital.

**Results:** Glutathione S-transferase alpha 1 (GSTA1) was identified as a driver of neuroendocrine differentiation in PCa cells using DSP sequencing of tissue microarrays prepared from clinical samples. Following enzalutamide (ENZ) treatment, GSTA1 expression is inhibited. Decreased GSTA1 levels have also been reported in patients with neuroendocrine PCa (NEPC). GSTA1 knockdown leads to increased intracellular reactive oxygen species (ROS), which can activate the inflammatory gene, tumor necrosis factor receptor superfamily member 13B (TNFRSF13B). TNFRSF13B induces c-Fos expression, forming a transcriptional complex with c-Jun, thereby regulating chromogranin A (CHGA) and promoting the neuroendocrine phenotype.

**Conclusion:** Our study suggested that GSTA1 deficiency leads to elevated ROS levels and activation of TNFRSF13B and c-FOS, which subsequently transcriptionally regulate CHGA and ultimately drive neuroendocrine differentiation in PCa.

## Introduction

Neuroendocrine prostate cancer (NEPC) is a late manifestation of prostate cancer (PCa), regarded as a hormone-refractory subtype 1, 2. NEPC is gradually being considered as a subset of castration-resistant prostate cancer (CRPC), overcoming dependence on the AR pathway and acquiring the histological characteristics of neuroendocrine differentiation 3, 4. Although the application of next-generation androgen receptor inhibitors such as abiraterone and enzalutamide (ENZ) has successfully improved the survival rate of patients with advanced PCa, these treatments have also resulted in an increased incidence of NEPC 5, 6. Notably, the median overall survival of patients with NEPC is less than 1 year owing to the limited treatment options available 7. Additionally, it is currently difficult to understand the molecular basis of NEPC, which hinders the development of effective therapeutic strategies.

Compared to prostate adenocarcinoma, NEPC exhibits a loss of RB1 and TP53 and amplification of MYCN and AURKA 8-10. Neuroendocrine tumors are characterized by high neuroendocrine biomarkers, low AR signaling, and specific genomic and epigenetic features 11, 12. Prolonged inhibition of the AR pathway can lead to epithelial-mesenchymal transition (EMT) and neuroendocrine differentiation 13-15. Owing to the heterogeneity of NEPC and its unclear mechanisms of progression, targeting individual signaling molecules may have limited therapeutic efficacy. In contrast, inflammation promotes the malignant progression of tumors 16. Therefore, further elucidation of the effects of inflammation on cellular plasticity and treatment resistance in NEPC may provide potential therapeutic avenues for this highly aggressive disease.

c-FOS is a regulatory factor that is involved in cell proliferation and transformation. The expression of c-Fos is regulated by various enhancers such as cAMP response elements, serum response elements, and sis-inducible enhancers 17, 18. As part of Activator Protein 1 (AP-1), the binding of c-Fos to gene promoters is typically linked to transcriptional activation. Activated AP-1 (c-FOS/c-JUN) binds to promoter or enhancer regions, thereby regulating the transcription of downstream target genes 19. Various stimuli can activate AP-1, such as extracellular growth factors, oxidative stress, pro-inflammatory cytokines, interferons, and the intracellular PI3K/Akt and MAPK signaling pathways 19. Overexpression of AP-1 has been observed in numerous tumors, including triple-negative breast cancer, colon cancer, and anaplastic large cell lymphoma 20, 21, and it has been investigated as a potential therapeutic target.

Previous studies utilizing sequenced NEPC samples have been compromised by impurities, including contamination with adenocarcinoma cells or other cells. To overcome this, we specifically selected NEPC samples to obtain accurate information using digital spatial profiling (DSP). According to our data, neuroendocrine differentiation in PCa may be caused by the lack of glutathione S-transferase alpha 1 (GSTA1). Mechanistically, previous studies have suggested that GSTA1 deficiency activates TNFRSF13B and c-FOS through the induction of reactive oxygen species (ROS) elevation, further transcriptionally regulating CHGA to produce a neuroendocrine phenotype.

## Methods

### Cell culture

PCa cell lines LNCaP, 22RV1, DU145, PC3, and 293T were purchased from the Chinese Academy of Sciences, Shanghai Academy of Sciences, and C4-2 was purchased from the American Type Culture Collection (ATCC). All the cell lines were subjected to Short Tandem Repeat (STR) identification (Cellcook Biotech, Guangzhou, China). PCa cells were cultured in RPMI 1640 medium containing 10% fetal bovine serum (FBS), and 293T cells were cultured in DMEM medium containing 10% FBS. All complete culture media contained 100 U/mL penicillin and 100 µg/mL streptomycin. Cells were cultured in a damp 37 °C cell culture incubator containing 5% carbon dioxide. ENZ and the ROS inhibitor N-acetylcysteine (NAC) were purchased from MCE (Shanghai, China), and H_2_O_2_ was purchased from Nanguo Pharmaceutical (Guangzhou, China).

### RNA interference, lentivirus transduction, and plasmid constructs

Small interfering RNAs (siRNAs) targeting *GSTA1*, *TNFRSF13B*, and *FOS* were produced by GenePharma (Shanghai, China) and transfected using an siRNA transfection reagent. Lentivirus packaging *GSTA1* short hairpin RNAs (shRNAs) were synthesized by GenePharma. LNCaP and C4-2 cells were infected with lentiviral vectors and treated with puromycin for 7 days. The plasmids, including GSTA1, c-FOS, c-JUN, and luciferase reporter plasmids (pGL3-CHGA WT, pGL3-CHGA MUT1, pGL3-CHGA MUT2, pGL3-CHGA MUT3, and pGL3-CHGA MUT4) were purchased from YouBio (Hunan, China). To generate each lentivirus, 4.5 μg of pLP1, 2.25 μg of pLP2, and 2.25 μg of pLP/VSVG plasmids, along with 4.5 μg of GSTA1 plasmids, were transfected into 293T cells with 70-80% confluence in a 10-cm plate using Lipofectamine 2000 (Invitrogen).

### CCK8 proliferation assay

Cells were digested and diluted to 3000 per well, and 100 μL was seeded into the 96-well plate. The CCK8 reagent was added at 0, 24, 48, 72, and 96 h after cell incubation for 2 h, followed by the detection of OD 450 using an enzyme marker. The experiment was conducted three times, and the results were documented and analyzed.

### Sphere formation assay

DMEM/F12 medium devoid of FBS was prepared and supplemented with 10 ng/mL bFGF (Peprotech), 10 ng/mL EGF (Peprotech), and 1 ng/mL B-27 (Peprotech). A total of 1250 cells were seeded in ultra-low-attachment 24-well plates and supplemented with sphere assay-specific medium (0.5 mL). The edges of the 24-well plate were gently tapped around to distribute the cells evenly, followed by culture at 37 °C for 7-14 days in the cell culture incubator. Cell sphere formation was examined under a microscope, and the number of spheres with diameters exceeding 50 μm was recorded.

### Colony-forming assay

Five hundred cells from each well were seeded in a six-well plate and cultivated for 1-2 weeks. The cultures were monitored regularly, and once visible colonies emerged, the cultures were terminated. The cells were rinsed twice with PBS, fixed with 4% paraformaldehyde for 15 min, and subsequently stained with 0.1% crystal violet for 10 min. Colonies were observed under a microscope.

### Hematein-eosin (H&E) and immunohistochemistry (IHC)

The tissue fixed with paraformaldehyde was cut into 3-μm slices and attached to highly adhesive slides. Subsequently, the slides were placed at 75 °C for 2 hours, then dewaxed in xylene for 3 minutes three times, and placed in 100, 90, 80, and 70% ethanol solutions for 2 minutes each for rehydration. For H&E staining, the slides were stained with H&E for 1 min each. IHC was performed using antibodies against GSTA1 (1:400, Proteintech, China), CHGA (1:400, Finetest, China), and c-FOS (1:1000, Abcam, USA). Finally, detection was performed using a DAB Detection Kit (ZsBio, Beijing, China). Data were obtained using Leica Microsystems and Leica Application Suite v 4.12.0.

### Digital spatial profiling

Patients with PCa who underwent neoadjuvant therapy before radical prostatectomy were recruited from Nanjing Drum Tower Hospital. Following H&E and IHC staining, five patients exhibiting neuroendocrine differentiation post-treatment were identified. Two samples each of normal prostate, prostate adenocarcinoma, and NEPC were taken from the pathological wax blocks to prepare tissue microarrays for DSP sequencing using CapitalBio Technology. All procedures were performed in accordance with the Declaration of Helsinki and were approved by the Ethics Committee of Nanjing Drum Tower Hospital. For spatial samples, 47 rectangular regions of interest (ROIs) were designated based on the assessments by two pathologists. A segmentation mask was used to identify CK^+^ and SYP^+^ areas of interest (AOIs). Upon approval of the 47 ROIs, GeoMx DSP photocleaved the UV-cleavable barcoded linker of the bound RNA probes and collected individual segmented regions into separate wells in the DSP collection plate. DSP sequencing data were processed using the GeoMx NGS Pipeline.

### RNA sequencing

The RNA sequencing was conducted by LC-Bio Technology. Total RNA was extracted and purified using TRIzol reagent (Invitrogen, Carlsbad, CA, USA) following the manufacturer's procedure. RNA integrity was assessed using a biological analyzer 2100 (Agilent, California, USA), with an RIN value > 7.0, and was further confirmed by denatured agarose gel electrophoresis. Poly(A) RNA was purified from 1 μg total RNA using Dynabeads Oligo (dT) 25-61005 (Thermo Fisher Scientific, California, USA) through two rounds of purification. The average insertion length of the final cDNA library was 300 ± 50 bp. Finally, we performed 2×150 bp paired-end sequencing (PE150) on an Illumina NovaSeq 6000 (LC-Bio Technology, Hangzhou, China) using the vendor's recommended protocol.

### Reactive oxygen species (ROS) assay

Cells treated with or without NAC (10 mM) for 24 h were resuspended in diluted DCFH-DA (Beyotime, Shanghai, China) at a ratio of 1:1000. After incubation at 37 ºC for 20 min, ROS were detected by flow cytometry.

### Luciferase assay

According to the JASPAR prediction, different mutants containing the CHGA promoter were inserted into the pGL3-Basic Vector (YouBio). pGL3-derived plasmids (pGL3-CHGA WT, pGL3-CHGA MUT1, pGL3-CHGA MUT2, pGL3-CHGA MUT3, and pGL3-CHGA MUT4) and plasmids including c-FOS and c-JUN (YouBio) were co-transfected into 293T cells using Lipofectamine 8000. A Firefly Luciferase Reporter Gene Assay Kit (Beyotime) was used to detect luciferase activity.

### RNA isolation and RT-qPCR

Total RNA was extracted using TRIzol reagent. cDNA was synthesized using the PrimeScript RT Master Mix. ChamQ Universal SYBR qPCR Master Mix was used in conjunction with a StepOne Real-Time PCR System (Thermo Fisher Scientific, MA, USA). All experiments were conducted according to the manufacturer's guidelines. Data acquisition was conducted using QuantStudio 6 Flex Software version 1.3. Fold-changes in gene expression were determined using the comparative Ct method, and each cDNA sample was tested in triplicate. ACTB served as an internal reference gene for this analysis.

### Tumorigenicity assay in mice

PC3 cells transfected with the empty vector or the GSTA1 expression vector were digested, counted, and placed on ice. Male nude mice aged 4-5 weeks were immobilized and disinfected using alcohol swabs. Cells (5.5×10^6^ per mouse) were subcutaneously injected into nude mice. Survival status was observed, and tumor size was measured every other day. After 4-6 weeks, the mice were euthanized, and tumor tissues were collected. All animal experiments adhered to the ARRIVE guidelines and were conducted in accordance with the U.K. Animals (Scientific Procedures) Act of 1986, the EU Directive 2010/63/EU for animal experiments, and the National Research Council's Guide for the Care and Use of Laboratory Animals.

### Western blotting

RIPA buffer containing protease and phosphatase inhibitors was used to prepare the total cell extracts. The protein concentration of the samples was determined using a BCA Protein Quantification Kit Box 2 (Vazyme, China). Proteins were separated by sodium dodecyl sulfate-polyacrylamide gel electrophoresis (SDS-PAGE) at a constant pressure of 120 V and transferred to a PVDF membrane at a constant current of 300 mA. After blocking with 5% skim milk, the PVDF membranes were incubated with specific primary antibodies against GSTA1 (1:2000, Proteintech), SYP (1:2000, Proteintech), CHGA (1:2000, Finetest, China), AR (1:200, Santa Cruz, USA), TNFRSF13B (1:1000, Proteintech), c-FOS (1:1000, CST, USA), and p-FOS (1:1000, CST), respectively. After washing with PBST, the membranes were incubated with the secondary antibodies. Protein signals were then detected using an enhanced chemiluminescence (ECL) system (Tanon, Shanghai, China).

### Immunoprecipitation (IP)

The cells were harvested and lysed in IP lysis buffer containing protease and phosphatase inhibitors. Specific antibodies against the target proteins (c-JUN (CST) or c-FOS (Abcam)) or normal immunoglobulin G (IgG) were added to the cell lysates, incubating overnight at 4 °C. Protein G magnetic beads (CST) were added to the lysis buffer and incubated at room temperature for 20 min. The antibody-binding proteins captured by the magnetic beads were isolated and washed using a magnetic separation rack. After denaturation and heating at 100 °C for 5 min, the samples were used for immunoblotting experiments.

### Chromatin immunoprecipitation (ChIP)

For each ChIP reaction, 4×10^6^ cells were used. Most of the intracellular DNA was disrupted within 200-1000 bp by ultrasound. Subsequently, chromatin immunoprecipitation was performed using a c-FOS (CST) antibody. Please refer to the ChIP Detection Kit (CST) for more detailed information. Assessments were performed using gel electrophoresis and quantitative PCR (qPCR).

### Re-ChIP

The steps for sample sonication and chromatin immunoprecipitation were the same as those used for ChIP (c-FOS or c-JUN antibodies were added during chromatin immunoprecipitation). After incubation and washing with ChIP-grade Protein G Magnetic Beads, 25 μl of 10 mmol/L DTT was added and incubated at 37 °C for 30 minutes. After centrifugation, the supernatant was diluted 20-fold with re-ChIP buffer and divided into two tubes. Subsequently, c-JUN or c-FOS antibodies, as well as the IgG antibody, were added separately and incubated overnight at 4°C. The PCR amplification steps were the same as those used for ChIP.

### Statistical analysis

IBM SPSS Statistics 26 and GraphPad Prism 8 software were used for analysis and plotting. Quantitative data were presented as mean ± standard deviation (SD) and analyzed using Student's *t*-test. A p-value < 0.05 was considered a statistically significant difference.

### Sex as a biological variable

Our study exclusively examined male mice because the disease model is only relevant in males.

### Data availability

The gene expression profile data analyzed in the study were attained from the Gene Expression Omnibus under accession number GSE32967. Other data are available at https://www.cbioportal.org/. The expressed genes were downloaded from Gene Set Enrichment Analysis (GSEA) (https://www.gsea-msigdb.org/gsea/index.jsp). Additional raw data produced in this study are available from the corresponding author upon reasonable request.

## Results

### GSTA1 expression is downregulated in NEPC

**Figure 1:**
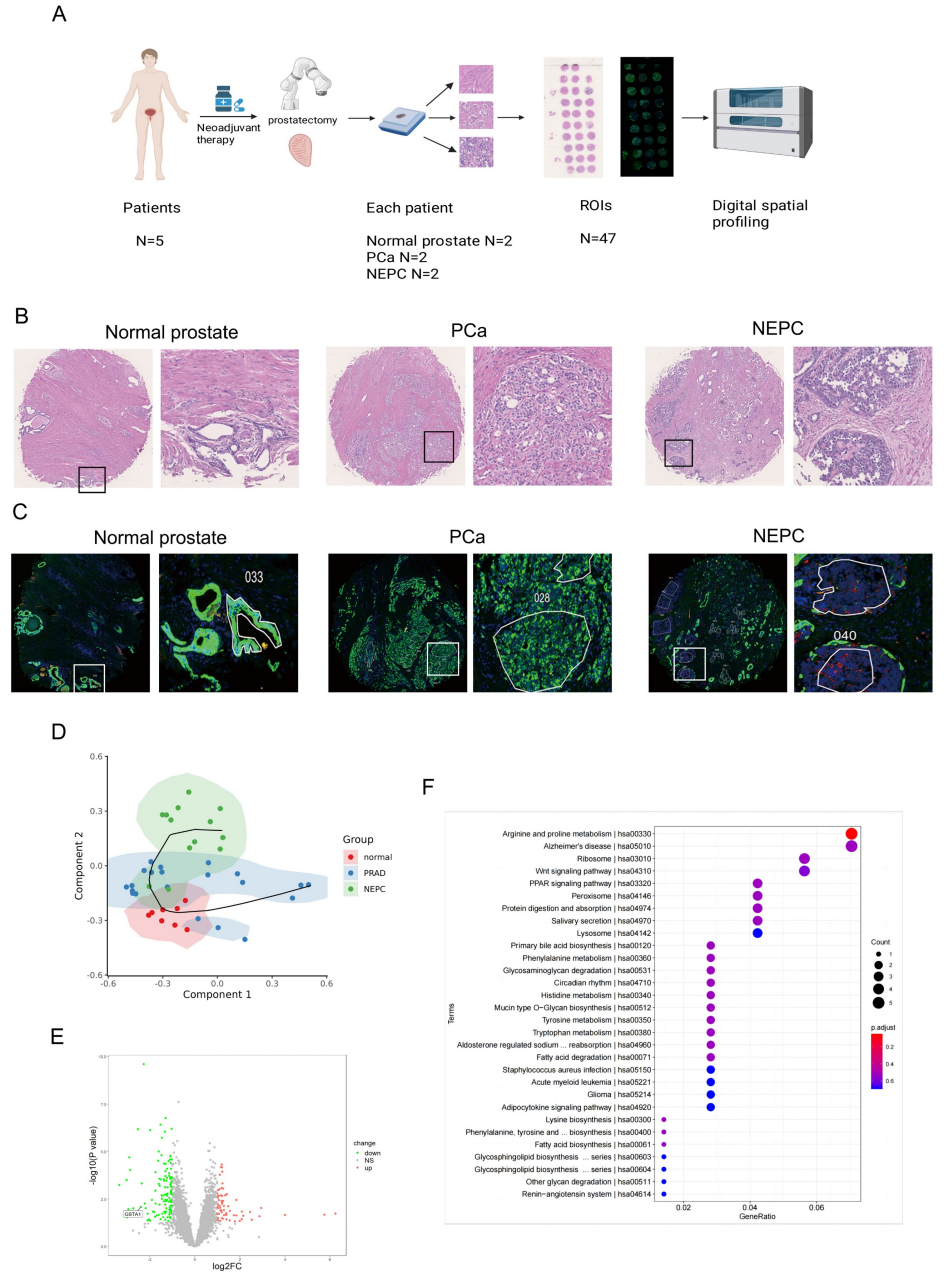
** Digital spatial profiling of paraffin-embedded NEPC.** (A) Five samples of PCa patients with neuroendocrine differentiation after drug treatment were selected. Normal prostate tissues, prostate adenocarcinoma tissues, and NEPC tissues of each sample were chosen to prepare tissue microarrays, with immunofluorescence staining and H & E staining. Scale bars, 500 μm (left), 250 μm (right). (B) Pathologic H & E staining of the patient and the circled regions of normal prostate, prostate adenocarcinoma, and NEPC subtypes. (C) Fluorescence staining of the patient and the circled regions of normal prostate, prostate adenocarcinoma, and NEPC subtypes. DAPI^+^ (Blue), PanCK^+^ (Green), SYP^+^ (Red). (D) The pseudotime analysis of the origin of NEPC in DSP sequencing. (E) Volcano plot of differentially expressed genes in the DSP sequencing. (F) KEGG pathway of prostate cancer and NEPC signature genes enrichment.

To explore the process of neuroendocrine differentiation in PCa, five patients who underwent hormone therapy and exhibited neuroendocrine differentiation were selected from the Drum Tower Hospital affiliated with Nanjing University. Two samples of normal prostate tissue, prostate adenocarcinoma tissue, and NEPC tissue were obtained from the surgical specimens of each patient to construct a tissue microarray (Fig. 1A). H&E staining revealed a deep-dyed nuclear morphology within the NEPC tissues (Fig. 1B). For further validation, immunohistochemical staining was performed on the tissue microarray, showing lower expression of CK and higher expression of SYP in NEPC tissues and higher expression of CK and lower expression of SYP in prostate adenocarcinoma tissues (Fig. S1A-B). Subsequently, immunofluorescent staining for DAPI (blue), CK (green), and SYP (red) was performed using the tissue microarray (Fig. 1C). Based on the immunofluorescence and H&E staining of adjacent continuous tissue slices, ROIs for analysis were accurately circled by pathologists.

The normal prostate is characterized by an orderly arrangement of small acini with a basal cell layer. The nucleus is located at the base, with delicate chromatin and indistinct nucleoli. In contrast, the CK-positive prostate adenocarcinoma samples demonstrated increased tumor cell density, enlarged and polymorphic cell nuclei, rough chromatin, significantly enlarged nucleoli, and disappearance of the basal cell layer. Meanwhile, the NEPC cells were observed to be round or oval, with uniform chromatin and irregular cell arrangements, commonly in nest-like, cord-like, or patchy shapes, without typical acinar structures.

**Figure 2:**
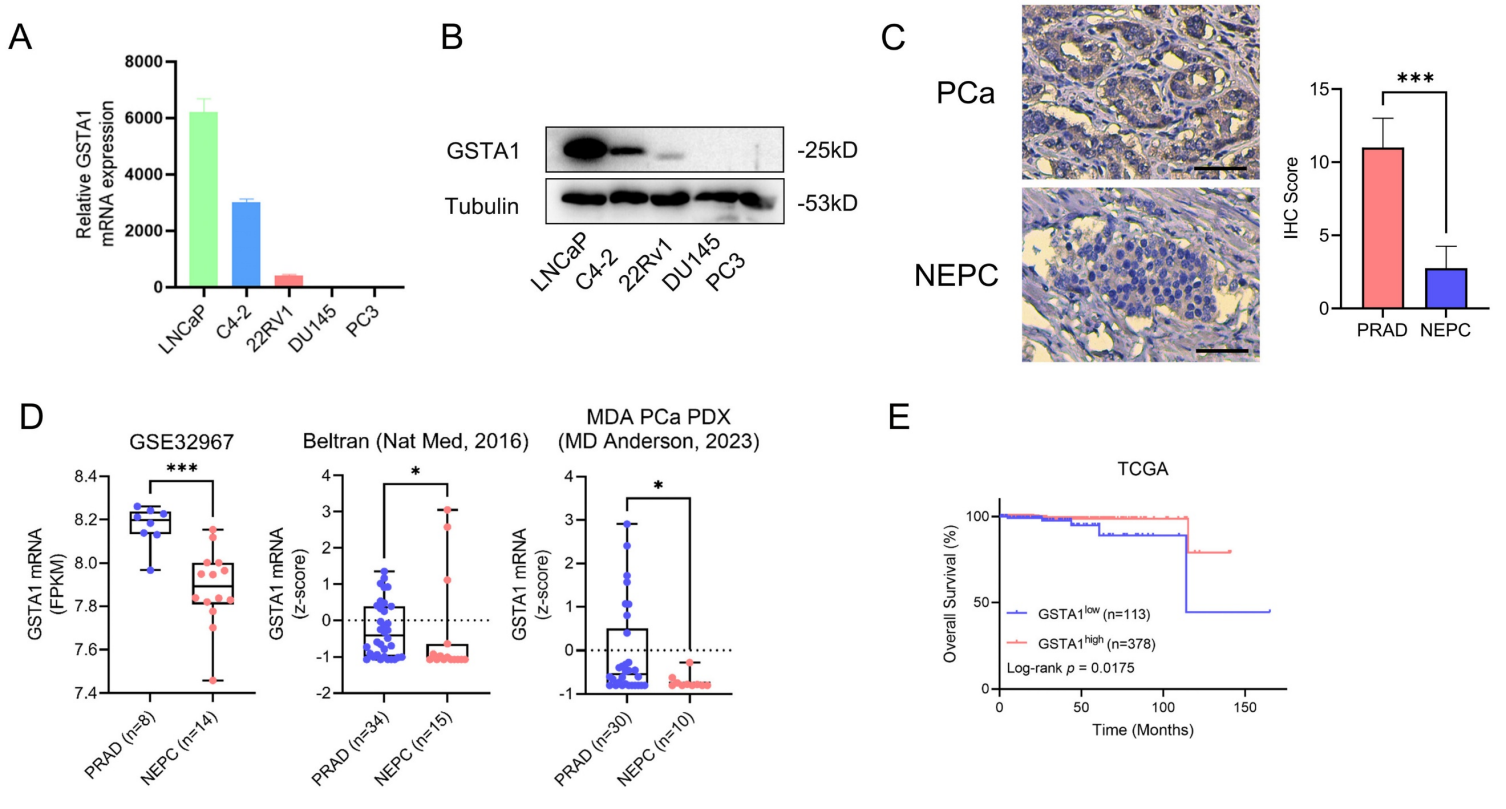
** The screening process of GSTA1 by DSP sequencing and its low expression in NEPC tissue and cells.** (A, B) GSTA1 in prostate cancer cell lines including LNCaP, C4-2, 22Rv1, DU145, and PC-3 by immunoblotting and RT-qPCR. (C) Immunohistochemical staining of GSTA1 in prostate adenocarcinoma and NEPC lesions from patients, scale bar: 250 μm. (D) mRNA levels of GSTA1 in prostate adenocarcinoma and NEPC samples. (E) OS of patients with stratified GSTA1 mRNA levels in the TCGA database.

In total, 8 ROIs of normal prostate tissues, 21 ROIs of prostate adenocarcinoma tissues, and 13 ROIs of NEPC tissues were selected for DSP sequencing. Pseudotime analysis using SCORPIUS was conducted to validate the trajectory of NEPC, suggesting that NEPC likely derives from prostate adenocarcinoma cells (Fig. 1D). The differentially expressed genes and Kyoto Encyclopedia of Genes and Genomes (KEGG) pathway enrichment of PCa and NEPC are presented (Fig. S1C, Fig. 1E-F). By analyzing the differentially expressed genes, we identified the top ten significantly upregulated and downregulated genes. The AR-related gene *KLK3* and NE-related genes *CHGA* and *CHGB* were excluded. The results of the CTPC and Rebecca database show that GSTA1 was highly expressed in AR positive cells such as LNCaP, C4-2, and 22RV1, and lowly expressed in NEPC cell lines H660 and LASCPC1, as well as AR negative cell lines PC3 and DU145 (Fig. S1D-E). Finally, 17 genes were tested in five PCa cell lines using RT-qPCR. The results revealed that *GSTA1* was highly expressed in the LNCaP and C4-2 prostate adenocarcinoma cell lines, whereas it was downregulated in the androgen-insensitive DU145 and PC3 NEPC cell lines, with this trend being the most pronounced (Fig. 2A-B, Fig. S1F). Notably, the differential expression levels of *TMPRSS2* and *FABP5* were slightly lower than those of *GSTA1*. For further validation, immunohistochemical staining for GSTA1 was performed on clinical specimens, showing notable staining in prostate adenocarcinoma and nearly absent staining in NEPC (Fig. 2C). Additionally, the NEPC dataset exhibited a significant decrease in GSTA1 expression compared to prostate adenocarcinoma, along with a negative correlation between GSTA1 and NEPC-related molecules 22, 23 (Fig. 2D, Fig. S1G-H). Importantly, PCa, with higher malignancy rates, is more likely to undergo neuroendocrine differentiation 11. Furthermore, patients with low GSTA1 levels exhibit poorer overall survival (OS) (Fig. 2E). Based on these findings, the downregulation of GSTA1 in NEPC suggests a functional role of GSTA1 in neuroendocrine differentiation.

### Androgen deprivation therapy (ADT)-induced loss of GSTA1 drives neuroendocrine differentiation in PCa

**Figure 3:**
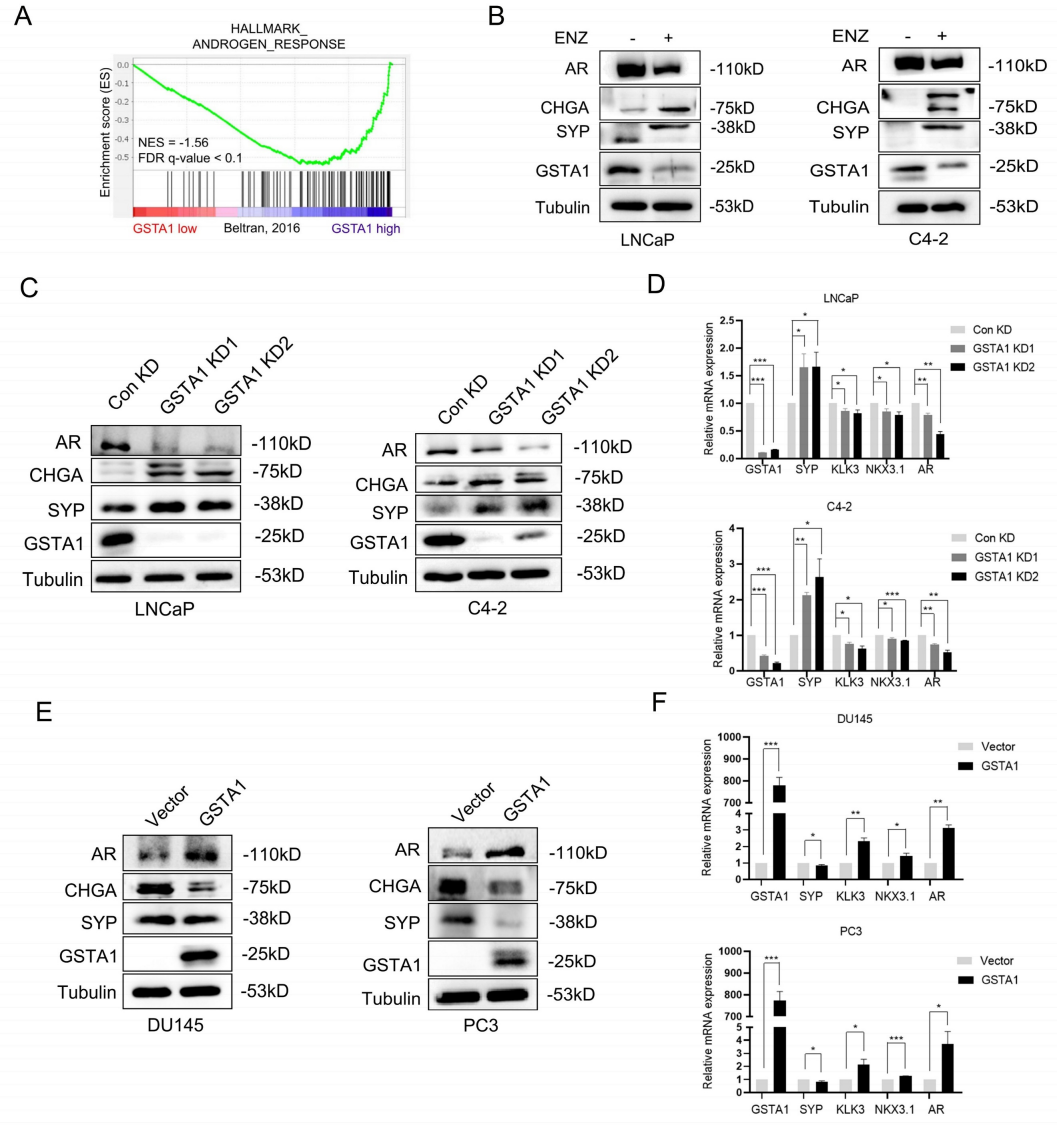
** ENZ inhibits GSTA1 to drive neuroendocrine differentiation in PCa.** (A) The correlation between the AR signaling pathway and the expression of GSTA1 by GSEA analysis. (B) After treatment with ENZ (10 μM) for 7 days, AR, CHGA, SYP, and GSTA1 were detected in LNCaP and C4-2 cells by immunoblotting. (C) AR, CHGA, SYP, and GSTA1 in control and GSTA1-silenced LNCaP and C4-2 cells by immunoblotting. (D) SYP, KLK3, NKX3.1, AR, and GSTA1 in control and GSTA1-silenced LNCaP and C4-2 cells by RT-qPCR. (E) AR, CHGA, SYP, and GSTA1 in control and GSTA1-overexpressing DU145 and PC3 cells by immunoblotting. (F) SYP, KLK3, NKX3.1, AR, and GSTA1 in control and GSTA1-overexpressing DU145 and PC3 cells by RT-qPCR. The bar graph data are presented as mean ± SD, * p < 0.05, ** p < 0.01, *** p < 0.001.

The emergence of NEPC was observed in clinical samples after treatment. To investigate the potential regulation of GSTA1 by ADT, the correlation between AR signaling pathways and GSTA1 was analyzed in the NEPC dataset using GSEA. The analysis showed a positive relationship between *GSTA1* expression and AR signaling pathways (Fig. 3A). ENZ treatment decreased the expression of GSTA1 and AR and increased the expression of CHGA and SYP in LNCaP and C4-2 cells, indicating that the AR antagonist facilitated the neuroendocrine differentiation process (Fig. 3B). *GSTA1* knockdown in AR-sensitive cells (LNCaP and C4-2) led to elevated expression of neuroendocrine markers (SYP and CHGA) and reduced expression of the AR-associated marker (AR) (Fig. 3C). Depletion of GSTA1 led to increased expression of neuroendocrine marker (SYP) and decreased AR-related marker (*KLK3, NKX3.1,* and *AR*) expression at the mRNA level (Fig. 3D). Conversely, overexpression of GSTA1 in AR-insensitive cells (DU145 and PC3) resulted in the opposite trend (Fig. 3E-F).

### GSTA1 knockdown induces malignant progression of PCa cells

**Figure 4:**
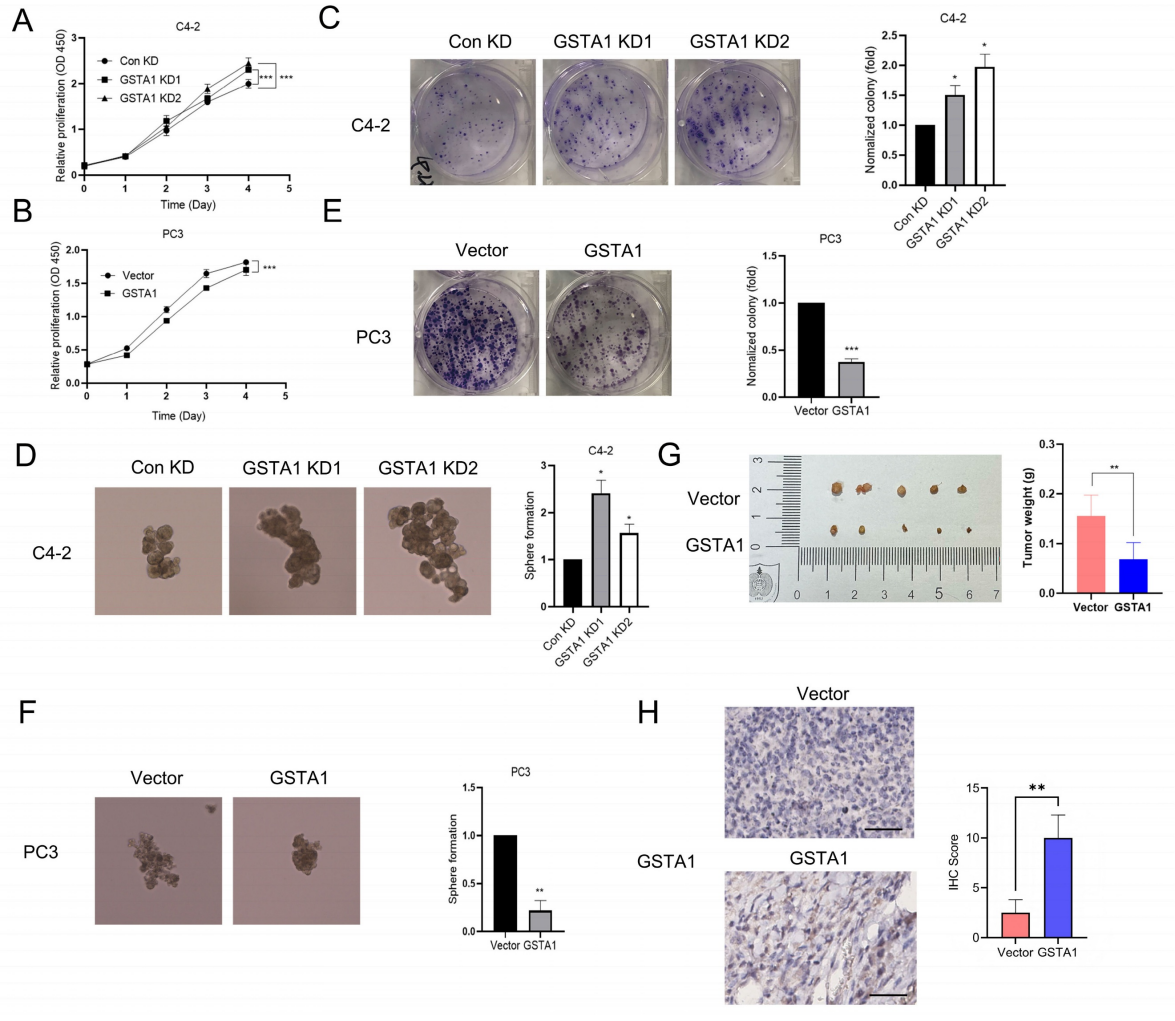
** GSTA1 deficiency induces malignant phenotype of PCa cells.** (A, B) Proliferation of control and GSTA1-silenced C4-2 cells and control and GSTA1-overexpressing PC3 cells. (C, D) Colony formation and sphere formation of control and GSTA1-silenced C4-2 cells. (E, F) Colony formation and sphere formation of control and GSTA1-overexpressing PC3 cells. (G) Images of tumor xenografts in male nude mice 4-6 weeks after subcutaneous inoculation with control and GSTA1-overexpressing PC3 cells. (H) IHC staining of subcutaneous tumors with GSTA1 in tumor-bearing mice. Scale bars 100 µm. Bar graphs display data as mean ± SD, * p < 0.05, ** p < 0.01, *** p < 0.001.

Previous studies have reported an association between neuroendocrine phenotypes and cellular stemness 24. To explore the influence of GSTA1 on the function of PCa cells, we generated C4-2 cells with stable GSTA1 knockdown and PC3 cells with stable GSTA1 overexpression. The results revealed that knockdown of GSTA1 significantly increased the proliferation rate of C4-2 cells, whereas GSTA1 overexpression significantly reduced the proliferation rate of PC3 cells (Fig. 4A-B). Additionally, C4-2 cells with GSTA1 knockdown exhibited increased colony and sphere formation (Fig. 4C-D, Fig. S2A), whereas PC3 cells overexpressing GSTA1 exhibited decreased colony and sphere formation (Fig. 4E-F). To explore the role of GSTA1 *in vivo*, we subcutaneously implanted nude mice with PC3 cells stably overexpressing GSTA1. Compared to control PC3 cells, mice injected with GSTA1- overexpressing PC3 cells showed a significant reduction in tumor formation (Fig. 4G). IHC analysis indicated an increase in GSTA1 levels in tumors from mice injected with GSTA1-overexpressing PC3 cells (Fig. 4H). These data suggest that GSTA1 deficiency induces tumor formation in PCa cells and promotes proliferation and stemness.

### GSTA1 deficiency activates the TNFRSF13B/c-FOS axis

**Figure 5:**
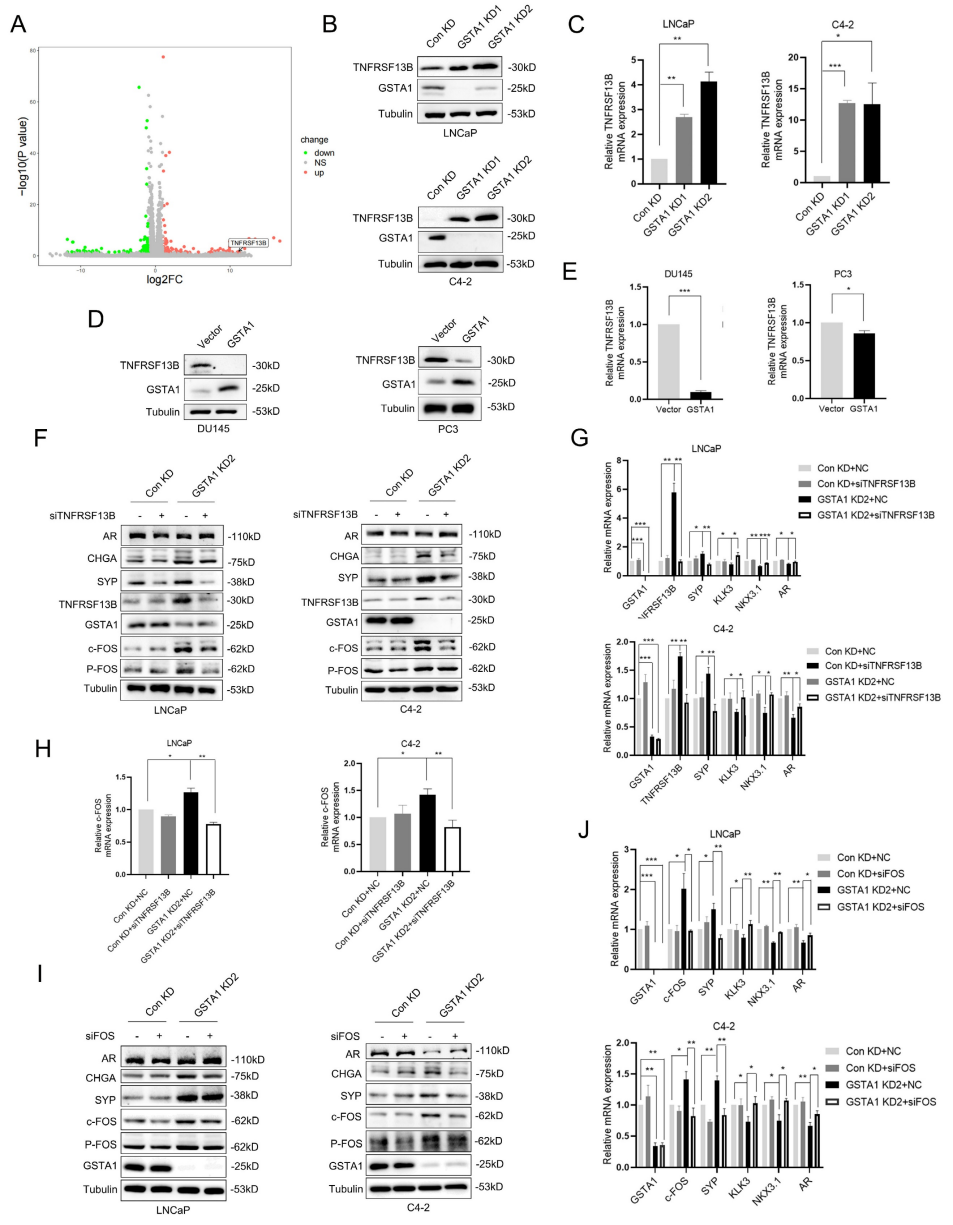
** The deficiency of GSTA1 activates the TNFRSF13B/c-FOS axis.** (A) Volcano plot of differential expressed genes in RNA sequencing data from stable GSTA1-knockdown C4-2 cells. (B, C) TNFRSF13B in control and GSTA1-silenced LNCaP and C4-2 cells using immunoblotting and RT-qPCR. (D, E) TNFRSF13B in control and GSTA1-overexpressing DU145 and PC3 cells using immunoblotting and RT-qPCR. (F) AR, CHGA, SYP, TNFRSF13B, GSTA1, c-Fos and p-Fos in control and GSTA1-silenced LNCaP and C4-2 cells after TNFRSF13B knockdown using immunoblotting. (G) SYP, KLK3, NKX3.1, AR, TNFRSF13B and GSTA1 in control and GSTA1-silenced LNCaP and C4-2 cells after TNFRSF13B knockdown using RT-qPCR. (H) The overall and phosphorylation levels of c-Fos in control and GSTA1-silenced LNCaP and C4-2 cells after TNFRSF13B knockdown using RT-qPCR. (I) AR, CHGA, SYP, c-Fos, p-Fos, and GSTA1 in control and GSTA1-silenced LNCaP and C4-2 cells after c-FOS knockdown using immunoblotting. (J) SYP, KLK3, NKX3.1, AR, c-Fos and GSTA1 in control and GSTA1-silenced LNCaP and C4-2 cells after c-FOS knockdown using RT-qPCR. Bar graphs display data as mean ± SD, * p < 0.05, ** p < 0.01, *** p < 0.001.

TNF is a crucial pro-inflammatory cytokine present in the tumor microenvironment, and its signaling is involved in tumor cell metastasis and acquired drug resistance 25. RNA sequencing analysis revealed that GSTA1 knockdown activated TNF signaling (Fig. S3A). Combined with the heatmap analysis, we hypothesized that GSTA1 may regulate the expression of TNFRSF13B (Fig. 5A). TNFRSF13B, a member of the TNF receptor superfamily, plays a role in cancer progression and affects cell proliferation via the p53 signaling pathway 26. It can also serve as a potential liquid biomarker for predicting cancer prognosis 27. Following GSTA1 knockdown, both the protein and RNA levels of TNFRSF13B were upregulated in LNCaP and C4-2 cells (Fig. 5B-C). Conversely, both the protein and RNA levels of TNFRSF13B decreased after GSTA1 overexpression in androgen-insensitive DU145 and PC3 cells (Fig. 5D-E). Furthermore, knockdown of TNFRSF13B in GSTA1-silenced LNCaP and C4-2 cells resulted in the downregulation of neuroendocrine markers and the upregulation of AR-related markers (Fig. 5F-G). TNFRSF13B knockdown was repeated in DU145 and PC3 cells (Fig. S3B-C). These findings suggest that GSTA1 may affect the cellular pathways involved in cancer progression by regulating TNFRSF13B.

TNFRSF13B has been confirmed to induce the activation of the transcription factor complex AP-1 28. RNA sequencing has also identified c-FOS as a potential downstream target of TNFRSF13B, which drives neuroendocrine differentiation (Fig. S3D). Notably, c-FOS plays a role in tumor progression by regulating inflammation and metabolism 29. In this study, the overall phosphorylation levels of c-Fos increased after GSTA1 knockdown but were reversed by TNFRSF13B knockdown in LNCaP and C4-2 cells (Fig. 5F, H). However, contrasting results were observed in DU145 and PC3 cells overexpressing GSTA1 (Fig. S3B, E). Following the confirmation of the regulation of c-Fos by TNFRSF13B, downregulation of neuroendocrine markers and upregulation of AR-related markers were observed in LNCaP and C4-2 cells after c-Fos knockdown (Fig. 5I-J). In addition, consistent trends were observed in the DU145 and PC3 cells (Fig. S3F-G). These findings indicate that GSTA1 deficiency activates the TNFRSF13B/c-FOS axis, leading to neuroendocrine differentiation in PCa.

### c-FOS and c-JUN transcriptionally activate CHGA

**Figure 6:**
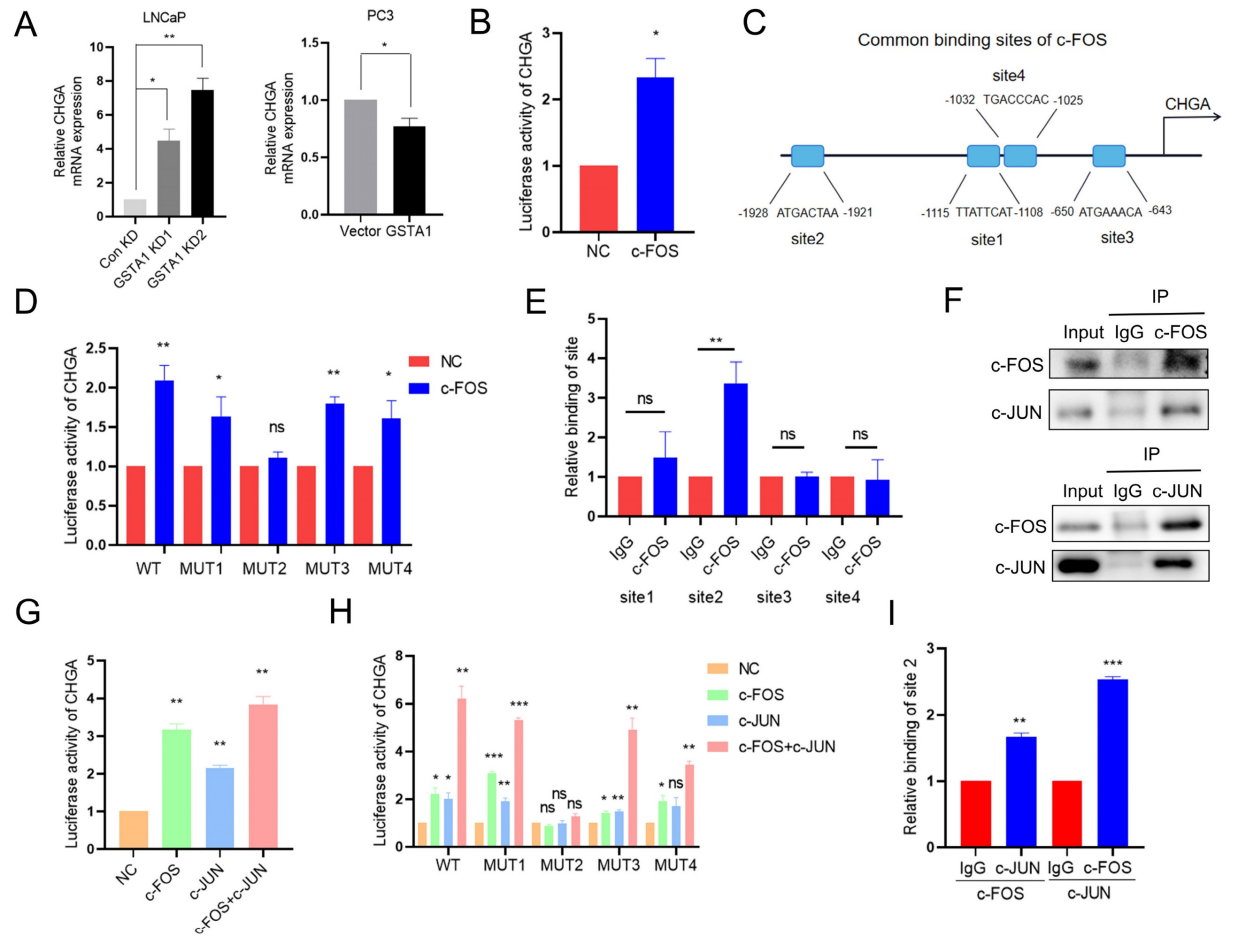
** c-FOS/c-JUN complex jointly transcriptionally regulates CHGA.** (A) CHGA in control and GSTA1-silenced LNCaP cells, control and GSTA1-overexpressing PC3 cells using RT-qPCR. (B) CHGA promoter reporter plasmids were co-transfected with c-FOS plasmids into 293T cells, and luciferase activity was detected after 48 hours. (C) Schematic diagram of potential c-FOS binding sites in the CHGA promoter region. (D) Mutated CHGA promoter reporter plasmids were co-transfected with c-FOS plasmids into 293T cells, and luciferase activity was detected after 48 hours. (E) ChIP experiment products were amplified using qPCR. (F) PC3 cell lysates were immunoprecipitated with c-FOS or c-JUN antibodies and the expression levels of c-Fos and c-Jun were detected using immunoblotting. (G) CHGA promoter reporter plasmids were co-transfected with c-FOS or c-JUN plasmids into 293T cells, and luciferase activity was detected after 48 hours. (H) Mutated CHGA promoter reporter plasmids were co-transfected with c-FOS or c-JUN plasmids into 293T cells, and luciferase activity was detected after 48 hours. (I) Re-ChIP experiment products were detected by qPCR. All experiments were carried out in triplicate. Data are presented as mean ± SD.

The potential role of c-FOS as a transcription factor in the regulation of neuroendocrine markers was previously investigated. It was predicted that c-FOS could transcriptionally regulate neuroendocrine markers such as *CHGA, SYP,* and *ENO2* based on JASPAR analysis. *CHGA* exhibited the most pronounced changes in both DSP and RNA sequencing analyses and was therefore selected for subsequent analysis. This glycoprotein is expressed in numerous neuroendocrine and neurons 30. By intersecting JASPAR predictions with RNA sequencing data, c-FOS was identified as a potential transcription factor for *CHGA* (Fig. S4A). Consistent with this prediction, *CHGA* was upregulated in LNCaP cells following GSTA1 knockdown, and downregulated in PC3 cells following GSTA1 overexpression (Fig. 6A). Subsequent luciferase assays also revealed that c-FOS enhanced the transcriptional activity of *CHGA* (Fig. 6B).

Next, the four highest-matching sites predicted for c-FOS in the *CHGA* promoter region were selected for further investigation (Fig. 6C). Interestingly, only mutated site 2 reversed the increase in luciferase activity induced by c-FOS (Fig. 6D). Consistently, ChIP experiments showed a significant increase in c-FOS binding to site 2 in the *CHGA* promoter region compared with the negative IgG control (Fig. 6E), supporting direct transcriptional regulation.

In addition to acting independently, c-FOS is known to dimerize with proteins from the c-JUN family to form AP-1, which plays a critical role in cell proliferation and transformation 31. Importantly, the predicted binding sites on the *CHGA* promoter are shared by c-FOS and c-JUN, suggesting the potential formation of a transcriptional complex between these two factors to co-regulate CHGA. Supporting this hypothesis, GSEA revealed a positive correlation between AP-1 family members and NEPC, although the difference was not statistically significant (Fig. S4B). Co-immunoprecipitation confirmed the interaction between c-Fos and c-Jun in PCa cells (Fig. 6F). Furthermore, the overall and phosphorylated levels of c-Jun were upregulated in LNCaP cells following GSTA1 knockdown, whereas they were downregulated in PC3 cells following GSTA1 overexpression (Fig. S4C).

Luciferase activity assays conducted on 293T cells showed that the overexpression of either c-Fos or c-Jun increased CHGA luciferase activity, with the highest increase observed when both factors were overexpressed together (Fig. 6G). Additionally, mutation of binding site 2 reversed the increased luciferase activity induced by the co-overexpression of c-Fos and c-Jun (Fig. 6H). Re-ChIP assays further confirmed the formation of a transcriptional complex at site 2 by c-Jun and c-Fos (Fig. 6I). Collectively, these results suggest that there is indeed a transcriptional complex formed by c-FOS and c-JUN that co-regulates CHGA expression in PCa.

### GSTA1 inhibits TNFRSF13B by regulating ROS levels

**Figure 7:**
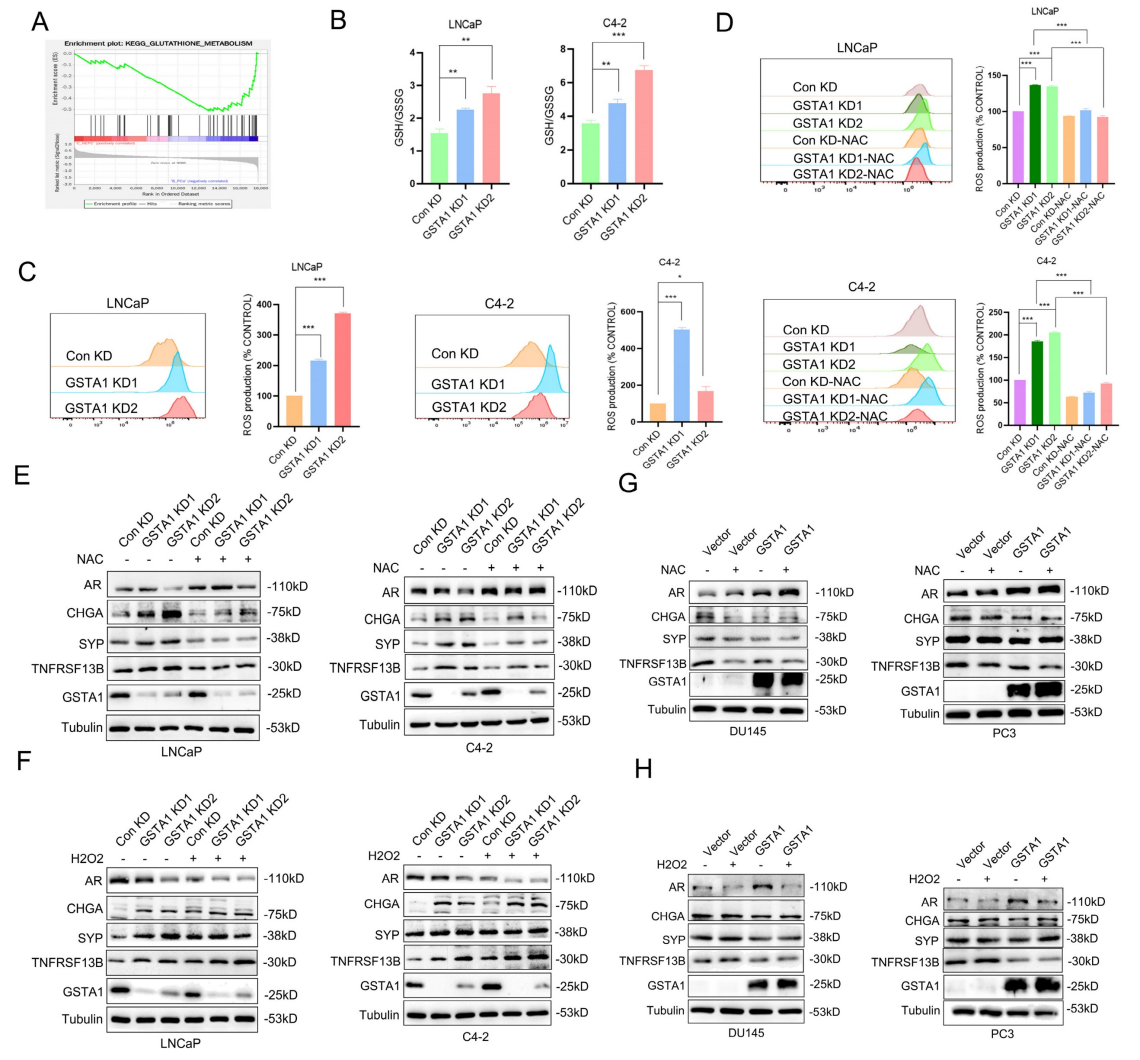
** The loss of GSTA1 restricts the function of GSH, leading to an increase in ROS levels and TNFRSF13B.** (A) GSEA of glutathione metabolism in the DSP dataset. (B) The GSH/GSSG ratio was detected in control and GSTA1-silenced LNCaP and C4-2 cells. (C) Flow cytometry analysis to measure ROS levels in control and GSTA1-silenced LNCaP and C4-2 cells, with statistical analysis of ROS levels shown in the histogram on the right. (D) Flow cytometry analysis of ROS levels after treatment with NAC (10 mM) for 24 hours in control and GSTA1-silenced LNCaP and C4-2 cells, with statistical analysis of ROS levels shown in the histogram on the right. (E) Immunoblotting for AR, CHGA, SYP, TNFRSF13B and GSTA1 after treatment with NAC (10 mM) for 24 hours in control and GSTA1-silenced LNCaP and C4-2 cells compared to the control group. (F) Immunoblotting for AR, CHGA, SYP, TNFRSF13B and GSTA1 after treatment with H_2_O_2_ (100 μM) for 24 hours in control and GSTA1-silenced LNCaP and C4-2 cells compared to the control group. (G) Immunoblotting for AR, CHGA, SYP, TNFRSF13B and GSTA1 after treatment with NAC (10 mM) for 24 hours in control and GSTA1-overexpressing DU145 and PC3 cells compared to the control group. (H) Immunoblotting for AR, CHGA, SYP, TNFRSF13B and GSTA1 after treatment with H_2_O_2_ (100 μM) for 24 hours in control and GSTA1-overexpressing DU145 and PC3 cells compared to the control group. The data shown in the histograms represent mean ± SD. * p < 0.05, ** p < 0.01, *** p < 0.001.

To elucidate the mechanism by which GSTA1 regulates TNFRSF13B in PCa, we analyzed enriched pathways involving GSTA1 using DSP sequencing data. In conjunction with the known function of GSTA1 and its enzyme family in protecting cells from ROS and peroxides, we observed inhibition of the glutathione metabolism pathway and glutathione transferase activity in NEPC (Fig. 7A, Fig. S5A-B). To validate the role of glutathione in GSTA1-related neuroendocrine differentiation, reduced glutathione (GSH) levels were measured following GSTA1 knockdown. The results showed an increased ratio of GSH to oxidized glutathione (GSSG) in LNCaP and C4-2 cells (Fig. 7B). This suggests that the loss of GSTA1 may impair the antioxidant effect of GSH, leading to its accumulation within cells and possibly causing oxidative stress. The upregulation of ROS levels was also observed in LNCaP and C4-2 cells after GSTA1 knockdown (Fig. 7C).

Treatment with the ROS inhibitor NAC for 24 h suppressed ROS levels (Fig. 7D). Similarly, ROS levels decreased in DU145 and PC3 cells overexpressing GSTA1 and were subsequently suppressed upon treatment with NAC (Fig. S5C-D), indicating that GSTA1 regulates ROS levels in PCa cells. Furthermore, AR-related markers were upregulated, whereas neuroendocrine markers were downregulated after treating GSTA1-knockdown LNCaP and C4-2 cells with NAC (Fig. 7E). In contrast, the treatment with H_2_O_2_ for 24 hours in LNCaP and C4-2 cells yielded opposite results (Fig. 7F), and similar results were observed in DU145 and PC3 cells overexpressing GSTA1 following treatment with NAC or H_2_O_2_ (Fig. 7G-H).

To further confirm the role of ROS in the upregulation of TNFRSF13B, *GSTA1*-knockdown LNCaP and C4-2 cells were treated with NAC or H_2_O_2_. Compared to the control, NAC suppressed the increase in TNFRSF13B, whereas H_2_O_2_ further promoted its elevation (Fig. 7E-F). Similar trends were observed in DU145 and PC3 cells overexpressing GSTA1 (Fig. 7G-H). These data suggested that GSTA1 regulates ROS levels to inhibit TNFRSF13B expression, thereby suppressing neuroendocrine differentiation in PCa.

### Human relevance of the TNFRSF13B/c-FOS/CHGA axis in NEPC

**Figure 8:**
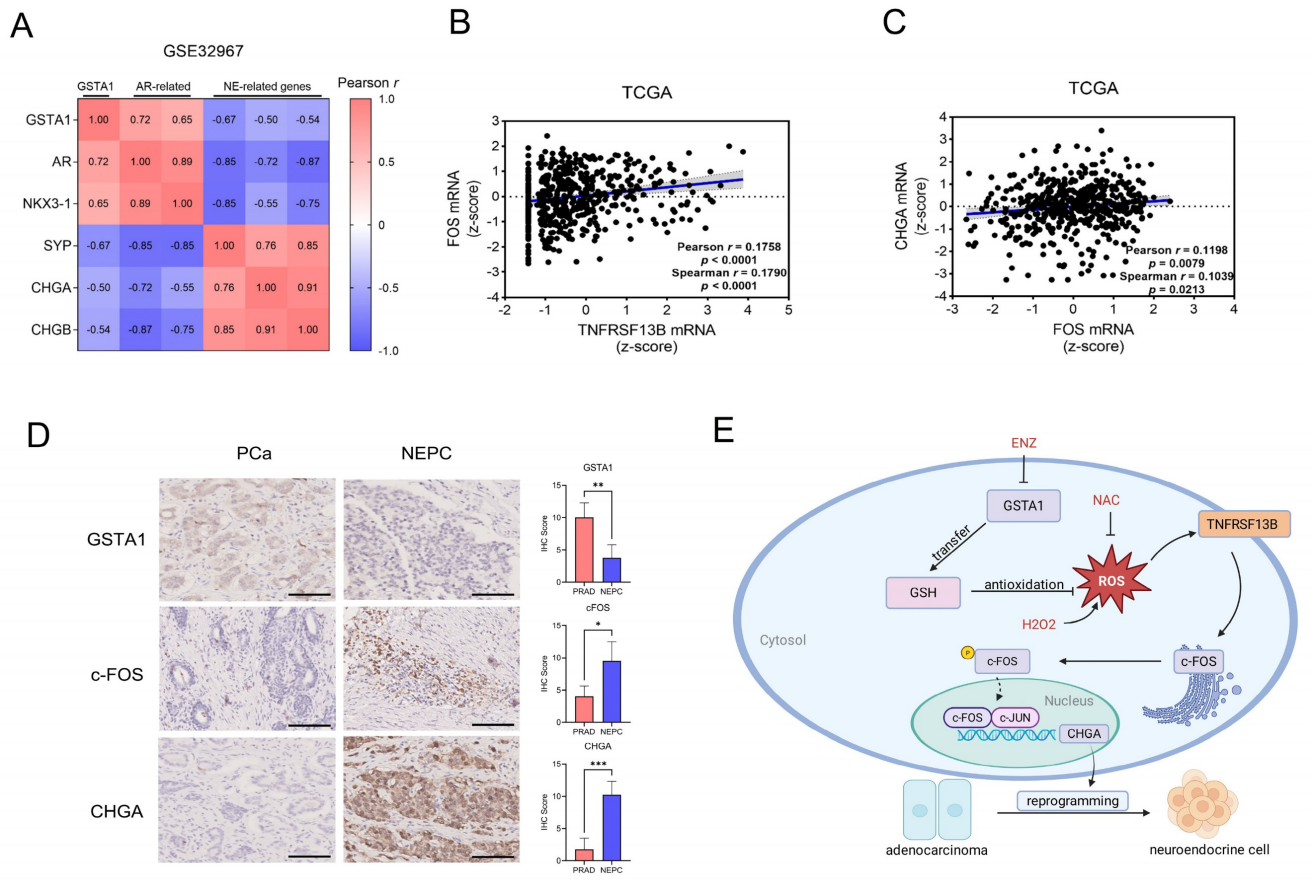
** Human Relevance of the TNFRSF13B/c-FOS/CHGA Axis in NEPC.** (A) Correlation analysis of GSTA1 with AR and neuroendocrine-related genes in the NEPC database. (B) mRNA correlation analysis of TNFRSF13B and c-FOS in the TCGA database. (C) mRNA correlation analysis of CHGA and c-FOS in the TCGA database. (D) IHC staining of GSTA1, c-FOS and CHGA of the tissue microarray from Nanjing Drum Tower Hospital. Scale bars, 250 μm. (E) Proposed model of the deficiency of GSTA1 induced by ADT leads to increased ROS levels and activation of TNFRSF13B, which activates c-FOS, forming a transcriptional complex with c-JUN, collectively regulating CHGA and driving neuroendocrine differentiation of PCa.

Bioinformatic analysis of human PCa and NEPC samples showed that GSTA1 was positively correlated with AR-related genes and negatively correlated with neuroendocrine-related genes (Fig. 8A, Fig. S6A). Analysis of gene expression in the TCGA PCa dataset revealed a positive correlation between *TNFRSF13B, FOS,* and *CHGA* (Fig. 8B-C). In our analysis, c-Fos was upregulated in NEPCs compared to PCa samples, although the difference was not statistically significant (Fig. S6B). However, immunohistochemical analysis of the tissue microarray collected from Nanjing Drum Tower Hospital showed decreased expression of GSTA1 and increased expression of c-Fos and CHGA in NEPC tissues relative to adenocarcinoma tissues (Fig. 8D). These results highlight the clinical relevance of the TNFRSF13B/c-FOS/CHGA axis driven by ADT-induced GSTA1 deficiency, which leads to neuroendocrine differentiation in PCa (Fig. 8E).

## Discussion

Patients with NEPC, a terminal subtype of PCa primarily induced by prolonged ADT, have a poor prognosis owing to the cancer's ability to develop treatment resistance through alternative lineage programs. The precise extraction of NEPC cells is complicated by their local occurrence and intermingling with adenocarcinoma in current NEPC-related research. To better understand the mechanisms of neuroendocrine differentiation and identify new clinical targets, DSP sequencing was employed to obtain accurate biological information from NEPC cells. Notably, pseudotemporal analysis of DSP sequencing indicated that NEPC may differentiate from prostate adenocarcinoma cells, providing theoretical support for studying neuroendocrine differentiation in PCa cells.

This study revealed that the loss of GSTA1 drives neuroendocrine differentiation in PCa. Notably, the GSTA1 enzyme family is essential for protecting cells from oxidative stress and peroxidative damage. Consistently, elevated levels of GSTA1 are correlated with extended overall survival and disease-free survival 32, whereas downregulation of GSTA1 occurs in poorly differentiated tumors, resulting in unfavorable clinical outcomes 33, which is consistent with our findings. Following ENZ treatment, GSTA1 expression was inhibited, leading to neuroendocrine-like characteristics via the inhibition of the AR signaling pathway. Thus, we speculated that the suppression of AR signaling after ADT may affect GSTA1, although our study did not further explore the upstream regulation of GSTA1. Supporting this notion, a previous study found that enhancing AR signaling (R1881) increased *GSTA1* mRNA expression and upregulated the expression of AR-related signaling molecules, whereas inhibition of AR signaling (knockdown of AR or addition of ENZ) showed the opposite trend. Moreover, after employing JASPAR to predict potential AR-binding sequences in GSTA1, we found relatively high scores, suggesting that AR directly regulates GSTA1 expression through transcriptional regulation. Consequently, GSTA1 deficiency caused the intracellular accumulation of GSH, leading to oxidative stress and increased ROS levels. Overall, our study provides novel insights into the role of GSTA1 in the progression of PCa.

An imbalance between ROS and the antioxidant defense system results in oxidative stress, leading to carcinogenesis 34. ROS are involved in the malignant transformation of cancer cells, and in the present study, sustained lineage transformation was observed at high ROS levels. While low levels of ROS exhibit beneficial effects by regulating intracellular signaling and homeostasis, high levels of ROS cause significant damage to proteins, lipids, and DNA, promoting carcinogenesis and other diseases 35. Importantly, ROS mediate responses to chemotherapy or radiotherapy by influencing downstream cell survival or death signaling cascades 36-38. These data indicate that ROS may be potential targets for combination therapy.

ROS serve as central regulators of inflammatory signaling, and their interactions with inflammation play a significant role in the pathogenesis of cancer. Our research revealed that elevated levels of ROS in the microenvironment activate the expression of TNFRSF13B, which in turn activates the transcription factor AP-1. Previous studies have demonstrated that TNFRSF13B plays a crucial role in humoral immunity by interacting with TNF ligands. It has also been implicated as an inflammatory response gene in the neuroendocrine differentiation of PCa. Furthermore, analysis based on public datasets showed that TNFRSF13B overexpression was associated with poor prognoses in patients with PCa. Notably, our study revealed the role of inflammation in the neuroendocrine differentiation of PCa; however, further investigation into the specific mechanism is warranted. For instance, GSTA1 indirectly regulates TNFRSF13B through ROS; however, this mechanism requires further comprehensive exploration. Supporting this notion, previous studies have shown that ROS can regulate the expression of BAFF 39, which then binds to and activates TNFRSF13B. This ROS-mediated BAFF expression is regulated by NF-κB activation, as increased intracellular oxidative stress promotes the activation of NF-κB 40. Once activated, NF-κB can bind to the BAFF promoter, leading to BAFF transcription and its subsequent production. Additionally, the ROS-p38 kinase-AP-1 cascade in LPS signaling has been suggested as another potential mechanism regulating BAFF 41.

c-FOS promotes EMT and cancer cell migration and invasion, exerting its effects through inflammation and metabolic regulation, as well as by acting as a regulatory factor in tumor progression through the promotion of EMT and reprogramming of cancer stem cells 29. Additionally, c-FOS participates in cell cycle regulation through cyclin A and acts as a direct or indirect transcriptional regulatory factor of cyclin D1 42. In our study, we found that TNFRSF13B expression was upregulated in the inflammatory microenvironment, leading to the activation of c-FOS, showing a positive correlation. Concurrently, c-FOS interacts with c-JUN in PCa cells. The AP-1 complex is rapidly activated by oxidative stress, and its activity is regulated by the phosphorylation of AP-1 proteins 43. These products form heterodimers via bZIP interactions for specific binding. Notably, c-FOS transcription and c-JUN phosphorylation primarily regulate the activity of AP-1 in T cells 44-46. AP-1 also participates in the malignant transformation of PCa by regulating genes related to metastasis, invasion, apoptosis, proliferation, and angiogenesis, thereby facilitating PCa progression 47-49. Upregulation of both c-JUN and c-FOS has been observed in advanced and metastatic PCa and is associated with disease recurrence and poor prognosis 50. CHGA, a sensitive and specific marker for diagnosing various types of neuroendocrine tumors 51, 52, was identified in our study as a direct transcriptional target of c-FOS in conjunction with c-JUN, thereby driving neuroendocrine differentiation in PCa cells. These results directly explain how GSTA1 deficiency drives neuroendocrine differentiation. In contrast, SYP and CHGA are expressed at very low levels in adenocarcinoma, corresponding with reduced FOS expression and low AP-1 complex activity. Therefore, we speculated that the binding in adenocarcinoma differs from that in NEPC, even for nuclear p-FOS and p-JUN under these conditions.

Our study provides a comprehensive understanding of the molecular mechanisms underlying lineage plasticity in PCa. We elucidated that the TNFRSF13B/c-FOS/CHGA pathway is responsible for driving the neuroendocrine differentiation of PCa through ADT-induced GSTA1 deficiency. Our findings not only have potential therapeutic implications in targeting GSTA1 but also suggest that ROS and the inflammatory microenvironment could serve as potential therapeutic targets for NEPC.

In conclusion, these data reveal the crucial role of GSTA1, a glutathione S-transferase, in the neuroendocrine differentiation of PCa. Experiments demonstrated that GSTA1 deficiency induced by ADT leads to elevated ROS levels and activation of TNFRSF13B. Subsequently, c-FOS, triggered by TNFRSF13B, forms a transcriptional complex with c-JUN, collectively regulating CHGA and driving the neuroendocrine differentiation of PCa. Our findings suggest that GSTA1 is a promising therapeutic target for NEPC.

## Supplementary Material

Supplementary figures and methods.
