# Supplementary material for: GSTA1 deficiency drives neuroendocrine differentiation via TNFRSF13B/c-FOS/CHGA axis in prostate cancer
Source: Int J Biol Sci. 2026 Jan 14;22(3):1632–47. doi: 10.7150/ijbs.120497 (PMC12839132; doi:10.7150/ijbs.120497)
Supplement: Supplementary file 1 — Supplementary figures and methods. [file ijbsv22p1632s1.pdf]

Fig. S1

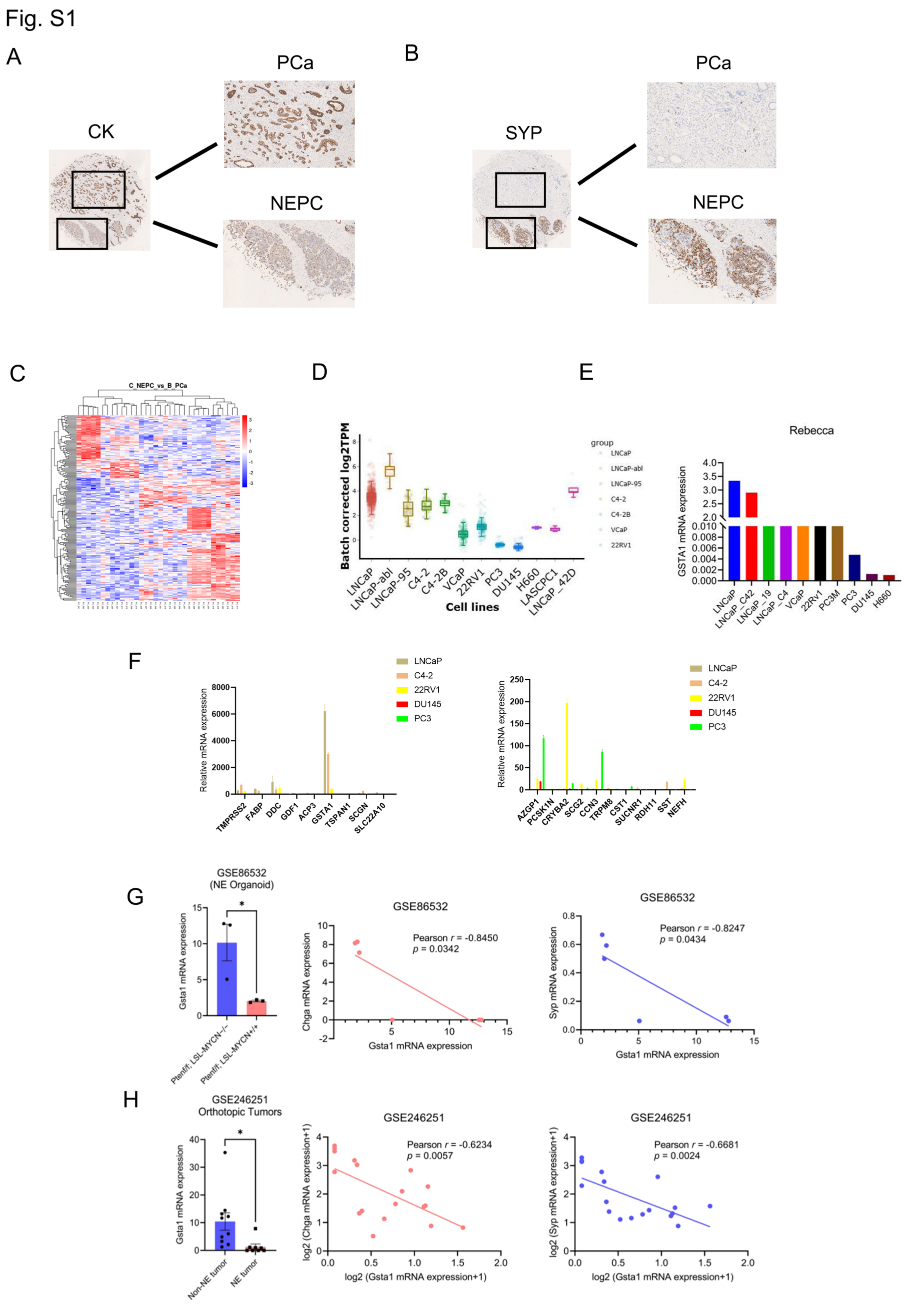

Figure S1. (A) IHC staining for CK in tissue microarray sections containing prostate cancer and NEPC tissues. Scale bars, 500 μm (left), 250 μm (right). (B) IHC staining for SYP in tissue microarray sections containing prostate cancer and NEPC tissues. Scale bars, 500 μm (left), 250 μm (right). (C) Heatmap of differentially expressed genes in DSP sequencing. (D) The expression of GSTA1 in different prostate cancer cell lines in the CTPC database. (E) The expression of GSTA1 in common prostate cancer cell lines in the Rebecca database. (F) RT-qPCR of selected differentially expressed genes in prostate cancer cell lines including LNCaP, C4-2, 22RV1, DU145,

PC3, with bar graphs displaying data as mean ± SD. (G) The expression of GSTA1 and correlation analysis with NE related molecules in the mouse NEPC organoid model. (H) The expression of GSTA1 and correlation analysis with NE related molecules In the mouse NEPC in situ tumor model.

Fig. S2

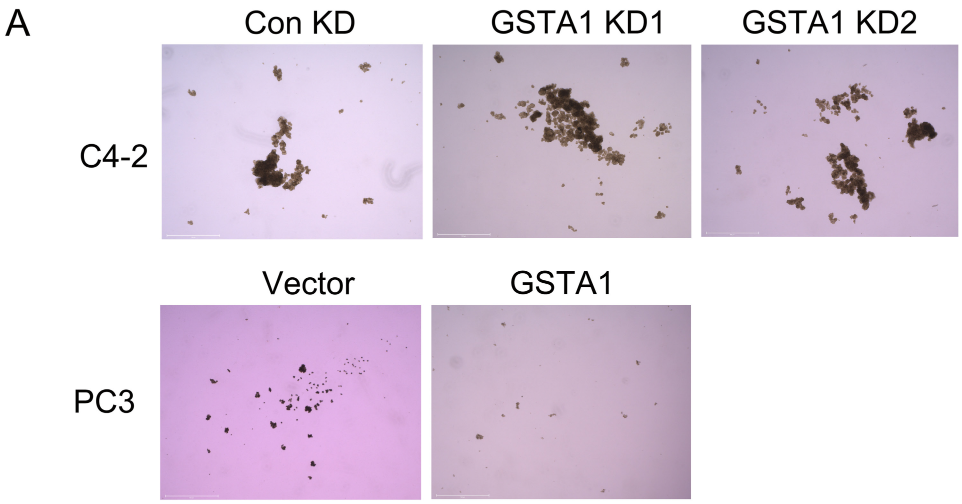

Figure S2. (A) Sphere formation of control and GSTA1-silenced C4-2 cells and GSTA1-overexpressing PC3 cells.

Fig. S3

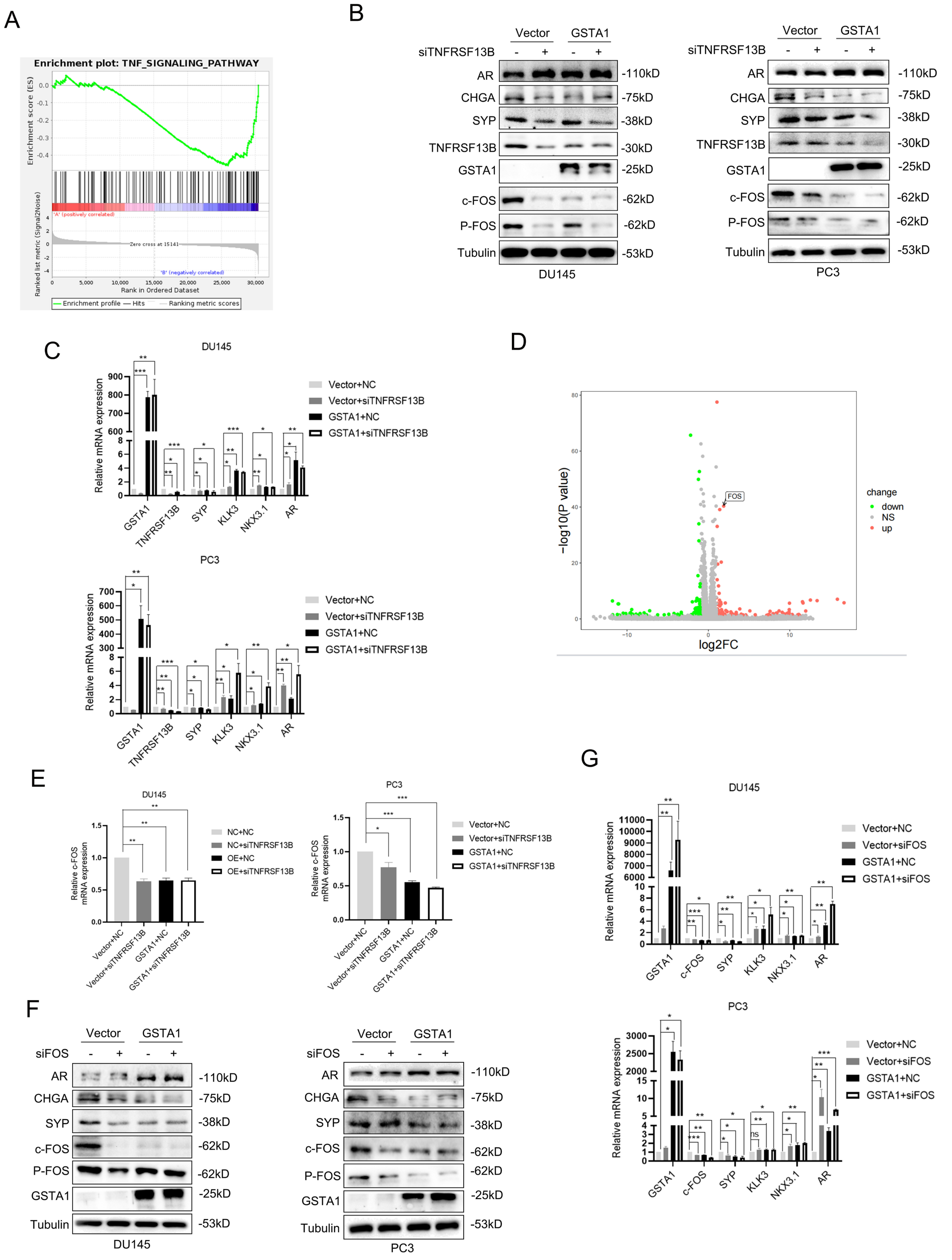

Figure S3. (A) GSEA analysis in RNA sequencing data from stable GSTA1-knockdown C4-2 cells. (B) AR, CHGA, SYP, TNFRSF13B, GSTA1, c-FOS and P-FOS in control and GSTA1-overexpressing DU145 and PC3 cells after TNFRSF13B knockdown using immunoblotting. (C) SYP, KLK3, NKX3.1, AR, TNFRSF13B and GSTA1 in control and GSTA1-overexpressing DU145 and PC3

cells after TNFRSF13B knockdown using RT-qPCR. (D) Volcano plot of differential expressed genes in RNA sequencing data from stable GSTA1-knockdown C4-2 cells. (E) The overall levels of c-FOS in control and GSTA1-overexpressing DU145 and PC3 cells after TNFRSF13B knockdown using RT-qPCR. (F) AR, CHGA, SYP, c-FOS, p-FOS, and GSTA1 in control and GSTA1-overexpressing DU145 and PC3 cells after c-FOS knockdown using immunoblotting. (G) SYP, KLK3, NKX3.1, AR, c-FOS and GSTA1 in control and GSTA1-overexpressing DU145 and PC3 cells after c-FOS knockdown using RT-qPCR. The bar graphs display data as mean ± SD, * $p < 0.05$, ** $p < 0.01$, *** $p < 0.001$.

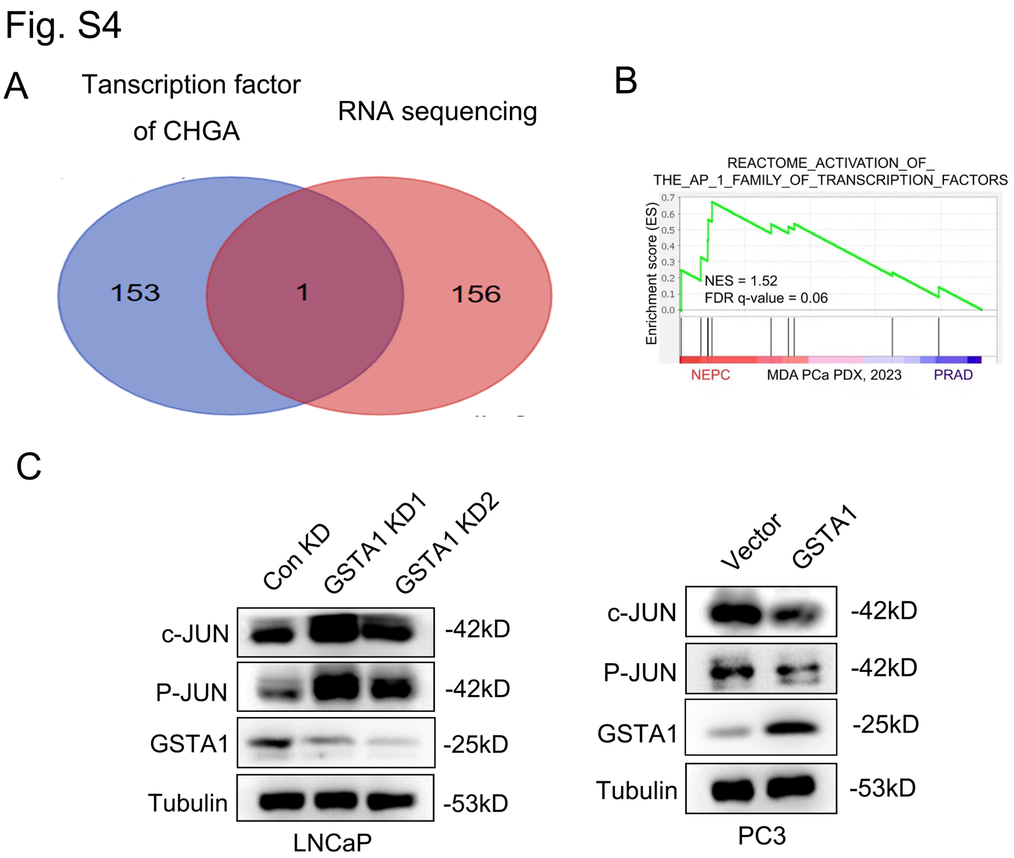

Figure S4. (A) Intersection of the predicted potential transcription factor set of CHGA and RNA sequencing. (B) GSEA of the AP-1 family transcription in the NEPC dataset. (C) The overall and phosphorylation levels of c-JUN in control and GSTA1-silenced LNCaP cells, control and GSTA1-overexpressing PC3 cells using immunoblotting.

Fig. S5

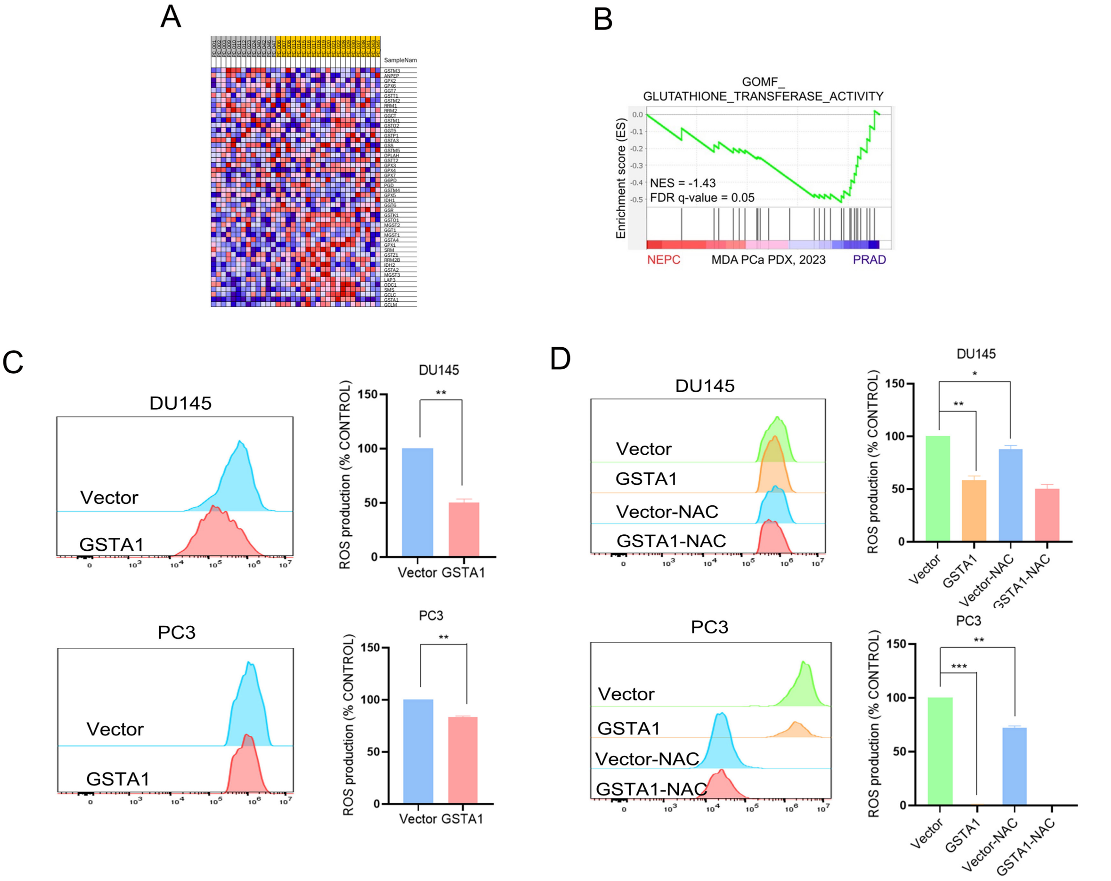

Figure S5. (A) Heatmap of the gene set for the glutathione metabolism pathway in DSP sequencing. (B) GSEA of glutathione transferase activity in the NEPC dataset. (C) ROS in control and GSTA1-overexpressing DU145 and PC3 cells using flow cytometry, with the histogram on the right showing the statistical analysis of ROS levels. (D) After treatment with NAC (10 mM) for 24 hours, ROS was detected in control and GSTA1-overexpressing DU145 and PC3 cells using flow cytometry in the treated and control groups, with the histogram on the right showing the statistical analysis of ROS levels. The bar graphs display data as mean ± SD, * $p < 0.05$, ** $p < 0.01$, *** $p < 0.001$.

Fig. S6

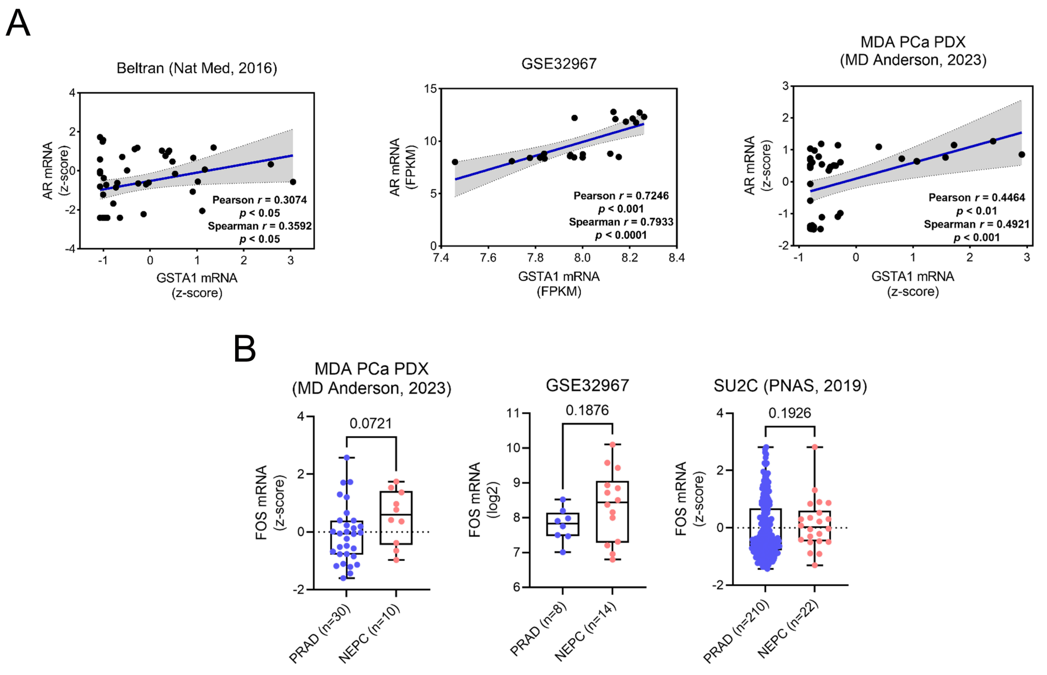

Figure S6. (A) mRNA correlation analysis of GSTA1 and AR in the published NEPC database. (B) mRNA levels of c-FOS in prostate adenocarcinoma and NEPC samples.

Methods

GeoMX Whole Transcriptome Atlas slide preparation

For the NanoString GeoMx DSP RNA assays, slides were prepared following the Manual RNA Slide Preparation Protocol in the GeoMx DSP Slide Preparation User Manual (NanoString, MAN-10115-05 for software v2.3). Briefly, slides were baked at 60°C for at least 30 minutes. Deparaffinization and rehydration were performed in citrisolv for 3x5 minutes, 100% ethanol for 2x5 minutes, 95% ethanol for 1x5 minutes and once in 1x PBS (Sigma Aldrich, P5368-10PAK) for 1 minute. Target retrieval was performed in 1x Tris EDTA pH 9.0 (eBioscience, 004956-58) in a Tinto Retriever Pressure Cooker (BioSB, BSB 7008) for 20 minutes at 99°C. Thereafter, the slides were washed 1x PBS for 5 minutes. Tissue RNA targets were exposed by incubating the slides with 1 ug/mL proteinase K (Ambion, 2546) in 1x PBS at 37°C for 15 minutes. Slides were washed in 1x PBS for 5 minutes, then immediately placed in 10% Neutral Buffered Formalin (EMS Diasum, 15740-04) for 5 minutes, followed by incubation in NBF Stop Buffer (1.48M Tris Base, 563mM Glycine) for 2x5 minutes, and once in 1x PBS for 5 minutes. The Whole Transcriptome Atlas (WTA) probe reagent (For Research Use Only) was diluted in pre-equilibrated buffer R to a final probe concentration of 4 nM and added to each slide, which was covered with a Hybrislip cover (Grace Bio-Labs) and incubated for 15 hours at 37°C in a HybEZ II System, humidified with 2× SSC (saline-sodium citrate) buffer. The following day, The HybriSlips were removed by dipping the slides in 2x SSC (Sigma Aldrich, S6639)/0.1% Tween20. To remove unbound probes, the slides were washed twice in 50% Formamide (ThermoFisher, AM9342)/2x SSC at 37°C for 25 minutes, followed by two washes in 2x SSC for 2 minutes. Slides were blocked in 200 µL Buffer W (NanoString), placed in a humidity chamber and incubated at room temperature for 30 minutes. The optimal dilution solution of morphology marker including DAPI, CK and SYP, and nuclear staining SYTO13 (Nanostring) were used for distinguishing various morphology . One at a time, slides were dried of excess Buffer W, set in a humidity chamber, covered with 200 µL morphology marker solution, and left to incubate at room temperature for 2 hours. Slides were washed twice with 2x SSC for

5 minutes and immediately loaded onto the NanoString DSP instrument.

GeoMx DSP instrument and ROI selection

For spatial samples, slides were loaded into the slide holder of the GeoMx DSP machine and secured by closing the slide tray clamp. Each slide was covered with 2 mL of buffer S and the underside of the slides cleaned with 70% ethanol before loading the slide tray into the DSP. The scanning areas were set to only scan the tissues and the tissue boundaries were determined by adjusting the x and y scanning areas and the sensitivity. Each slide was scanned with a 20x objective. 47 rectangular region of interests (ROIs) were placed based on selection and assessment by a pathologist per tissue. When applicable, a segmentation mask was applied to ROIs selecting CK+ regions and SYP+ areas of interest (AOI). Each channel threshold for segments was manually adjusted to maximize inclusion of the AOI signal areas and minimize non-signal areas. In addition, a general setting of erode 1-2 µm, N-dilate 2 µm, hole size 160 µm2 and particle size 50 was used. After approval of the 47 ROIs, the GeoMx DSP photocleaves the UV cleavable barcoded linker of the bound RNA probes and collects the individual segmented areas into separate wells in the DSP collection plate.

GeoMx RNA Illumina library preparation

DSP collection sample plates were dried down by sealing the plates with aeroseal and placed at room temperature overnight. Following resuspension of the wells with 10 µL of DEPC-treated water the plates were incubated for 10 minutes at room temperature before briefly spun down. 96-well PCR plates were prepared by mixing 2 µL PCR mix (NanoString), 4 µL of index primer mix (NanoString) and 4 µL of DSP sample. The following PCR program was used to amplify the Illumina sequencing compatible libraries; 37°C for 30 min, 50°C for 10 min, 95°C for 3 min, followed by 18 cycles of (95°C for 15 sec, 65°C for 1 min, 68°C for 30 sec), 68°C for 5 minutes and a final hold at 4°C. The indexed libraries were pooled with a 8-channel pipette by combining 4 µL per well from the 12 columns into 8-well strip tubes. The combined 48 µl pools in the 8-well strip were incubated with 58 µL AMPure XP beads (Beckman Coulter, A63880) (1.2x bead to sample ratio) for 5 min. The 8-well strip

was placed on a magnetic stand for 5 minutes before removal of the supernatant. The beads were then washed twice with 200 µl of 80% ethanol and dried for 1 minute before being eluted with 10 µL elution buffer (10mM Tris-HCl pH 8, 0.05% Tween-20). The 8 samples were combined into two pools of 40 µL each and incubated with 48 µL AMPure buffer (2.5M NaCl, 20% PEG 8000) for 5 min. Following magnetic incubation for 5 minutes and removal of supernatants the beads were washed twice with 200 µL of 80% ethanol and dried for 1 minute before eluted with 17 µL elution buffer. The two pools were combined to a final elute of 34 µL. Sequencing library size was assessed with a DNA high sensitivity bioanalyzer assay (Agilent Technologies). Library concentration was assessed with Qubit dsDNA high sensitivity assay (Thermo Fisher Scientific). Total target counts per DSP collection plate for sequencing were calculated from the total sampled areas (µm2) reported in the DSP generated Lab Worksheet. For CTA and WTA libraries, the target sequencing depths were 30 and 100 counts/µm2, respectively. Each library was diluted to 4-10 nM and combined 22 to reach the estimated counts/µm2 per library in the final pool. All sequencing libraries were generated with unique indexes allowing pooled sequencing. WTA libraries for sequencing with an Illumina NovaSeq 6000 platform at a loading concentration of 365 pM with 5% PhiX.. The sequencing parameters used were: read 1, 27 cycles; read 2, 27 cycles; index 1, 8 cycles; index 2, 8 cycles.

NanoString GeoMX data processing

DSP sequencing data were processed with the GeoMx NGS Pipeline (DND). After sequencing, reads were trimmed, merged, and aligned to a list of indexing oligos to identify the source probe. The unique molecular identifier (UMI) region of each read was used to remove PCR duplicates and duplicate reads, thus converting reads into digital counts.The limit of quantitation (LOQ) was estimated as the geometric mean of the negative control probes plus 2 geometric standard deviations of the negative control probes. Targets were removed that consistently fell below the LOQ, and the datasets were normalized using upper quartile (Q3) normalization.

Differential expression analysis

Comparisons between two groups were performed using unpaired two-tailed t-tests.

The multiple test correction of Benjamini-Hochberg was used to adjust the p-values of individual genes. The significant genes are defined as the fold chage > 2 and p-values <0.05.

Enrichment Analysis

GO enrichment, KEGG enrichment, Reactome enrichment and Disease enrichment (human only) of DEGs were performed using EnrichProfiler R-packages with Benjamini-Hochberg multiple testing adjustment,.The results were visualized using R package.
